# Who Would the Person Be after a Head Transplant? A Confucian Reflection

**DOI:** 10.1093/jmp/jhab024

**Published:** 2021-10-16

**Authors:** Lin Bian, Ruiping Fan

**Affiliations:** 1 Hebei Medical University, Shijiazhuang, PRC; 2 City University of Hong Kong, Hong Kong, PRC

**Keywords:** Confucianism, head transplant, familial relationalism, personal identity, qi

## Abstract

This essay draws on classical Confucian intellectual resources to argue that the person who emerges from a head transplant would be neither the person who provided the head, nor the person who provided the body, but a new, different person. We construct two types of argument to support this conclusion: one is based on the classical Confucian metaphysics of human life as qi activity; the other is grounded in the Confucian view of personal identity as being inseparable from one’s familial relations. These Confucian ideas provide a reasonable alternative to the currently dominant view that one’s personal identity “follows” one’s head. Together, these arguments imply that head transplantation is ethically inappropriate.

## I. INTRODUCTION: THE PERSONAL IDENTITY ISSUE ARISING FROM HEAD TRANSPLANTS

In December 2017, Italian doctor Sergio Canavero and his Chinese partner Xiaoping Ren announced that they had made a medical breakthrough and would soon perform the world’s first human head transplant in China. Head transplants, according to Canavero and Ren, were “imminent” (Hjelmgaard, 2017). Some American bioethicists immediately responded that this promise, for both scientific and ethical reasons, was truly “fake news” (Caplan, 2017). We called together a dozen Chinese scholars in the humanities to discuss relevant ethical and social issues. Our focus was not on technical problems. We fully understood that this medical “aspiration” still faced potentially intractable scientific challenges, such as how to avoid transplant rejection, how to provide continuous blood flow to the head, and how to connect the nervous systems of the head and the body. But we, as philosophers and ethicists, were intrigued to explore what head transplants, if technically possible, would mean for the nature of human life as well as for proper human relationships. In particular, we paid attention to the issue of personal identity: who would the person be after a head transplant (Fan et al., 2017)?

Most discussants in our group found this question baffling. However, some held that the person who survives such a surgery would be the person who provided the head (rather than the person who provided the body). In their view, one’s personal identity depends on the continuity of one’s mind (namely, one’s consciousness, memory, emotion, feeling, and the like), not on the intactness of one’s body. They held that modern science supported their view, since modern science, as they see it, has shown that the mind is generated by or pegged to the brain in the head. Accordingly, they did not object to head transplants in principle. They tended to believe that even if some scientific and technological innovations might be opposed initially by some “conservative” cultural forces, such developments cannot be permanently stopped. In their view, objections to head transplants grounded in concerns regarding personal identity are much like “playing God” objections against germline interventions: such worries or objections will gradually weaken and disappear.

The authors of this essay think that the personal identity issue raised by the possibility of head transplants should be taken seriously because this issue is intertwined with our bottom-line ethical considerations of the moral proprietary of head transplants. If the question of who the person is after head transplantation is unclear, we have no way to calculate the benefits or costs resulting from the procedure because we do not know whose benefits or costs are at stake. Moreover, if an individual’s personal identity is obscured, society cannot clearly distinguish one individual from another. Our fundamental moral concerns with individual dignity, liberty, and equality would become a nonstarter. Finally, as personal identity is correlated to the moral nature of human relations, we cannot reasonably explore proper human relations without carefully exploring concerns regarding personal identity.

In this essay, we draw on classical Confucian intellectual and moral resources to develop our arguments regarding the personal identity issues of head transplantation. We contend that the person who emerges from a head transplant would be neither the person who provided the head, nor the person who provided the body, but a new, different person. In particular, we construct two types of arguments to support our contention: one based on the classical Confucian account of human life as *qi* activity; the other grounded in the Confucian view of personal identity as being inseparable from one’s familial relations. The first type of argument is primarily metaphysical and the second social.

We understand that it is not a popular strategy to provide arguments grounded in traditional intellectual resources. However, we think it both acceptable and helpful to draw on traditional Confucian wisdom to tackle the challenges posed by contemporary technological advancements such as head transplants. First, the ultimate reality of the universe in general and the truth of human life in particular have not been exhausted by modern scientific theories. Plenty of room is left for traditional metaphysical and philosophical ideas, such as Confucian ones, for one to draw on to expand imagination in handling social and ethical issues. Moreover, scientific developments disclose puzzling phenomena, such as quantum entanglement, that not only call for further scientific exploration, but also acquiesce in the tenability of nonscientific accounts, such as the Confucian metaphysical account of *qi*, for one to use to comprehend the basic elements of the universe and human life. Indeed, classical Confucian metaphysical convictions transcend modern scientific theories and discoveries. They can broaden our philosophical scope, deepen our moral thought, and provide alternative perspectives to fashionable modern ideas. Finally, although the dominant political ideologies and institutions in East Asia are no longer Confucian, Confucian metaphysical and moral beliefs are still vibrant and embedded in the way of life lived by East Asian people. It is both helpful to these people as well as heuristic for others to disclose how Confucian tradition would locate the identity of the person after a head transplant. Accordingly, this study will hopefully shed light on the ethical implications of head transplants so as to decide whether it is morally appropriate to conduct head transplants, if they become technically possible.

## II. THE CHINESE IDEA OF *QI*: PHENOMENAL, METAPHYSICAL, AND MORAL

Some ancient civilizations, such as Greece and India, held an explicitly dualistic view of human life, which is usually called mind–body dualism. According to this view, human life is composed of two types of radically different and separable entities, mind and body, and mental activities are brought about not by bodily actions, but by immortal souls that occupy an independent realm of existence from that of the physical world. This view was philosophically refined by René Descartes (1596–1650), whose theory contends that the mind is a nonphysical (and therefore nonspatial) substance, whereas the body is a physical (and therefore extensional) substance. In contrast, Chinese tradition has never held such a dramatic dualist view (Qian, 1984). Since very ancient times, Chinese schools, such as Confucianism and Daoism, have embraced a uniquely monistic vision. This vision sees everything in the world in terms of *qi.*^1^

The original Chinese character of *qi* (氣) occurs in inscriptions on bones and tortoise shells as early as the sixteenth- through eleventh-century BCE. It shows the image of cloudy air, consisting of small, invisible airy elements (Gao and Lou, 2017). *Qi* was originally used to denote the tiny components of such natural phenomena as clouds and wind. This idea of *qi* (as small, invisible elements) developed into the metaphysical idea of *qi* (as the ultimate reality of myriad things in the world, including human life), which is conveyed in several Chinese foundational classics. One such classic is the *I Ching* (the Book of Changes), in which two types of *qi*, yin (陰) and yang (陽), are distinguished and are taken to be the two fundamental forces that form the deep structure of the universe. In particular, in this classic, the symbols of yin and yang (namely “--” for yin and “—” for yang) are used and combined to frame the Chinese metaphysical system of the eight diagrams and 64 hexagrams to show the changing states of affairs of the entire world (Legge, 1973). Another influential book that introduces the metaphysics of *qi* is the *Guanzi* (管子), recording the thought of a successful ancient Chinese administrator, Guanzhong (管仲, 719–645 BCE). In Chapter 49, the concept of essential *qi* (精氣 *jing qi*) is put forward:

This kind of *qi* is the essence of things, which brings about life. It generates five grains on earth, and forms various stars in heaven. Flowing between heaven and earth, it becomes ghosts and gods; stored in the human chest, it makes a holy person . . . This kind of *qi* cannot be controlled by force, but can be ensconced by virtue (德 *de*).^2^

The concept of essential *qi* implies a Chinese version of animism: organisms, objects, and places all possess a distinct spiritual essence, while human life carries more powerful essential *qi* than other animals or objects. In addition, after the concept of essential *qi* was proposed, the concept of original *qi* (元氣 *yuan qi*) was utilized to explain the origin of heaven and earth: original *qi* existed, developed, and formed heavenly objects, earthly materials, and a myriad of things in the world (Wang, 2017, 39). The original *qi* must all, or at least in part, be the essential *qi* so as to account for the animist view. In short, the Chinese concept of *qi* has developed into both phenomenal and metaphysical ideas. It has become a most elastic concept, carrying abundant denotations, including ideas of element, entity, substance, energy, medium, force, consciousness, spirit, and god (Zhang, 1996, 19–21).

Such diverse uses of *qi* are typical in Chinese texts and everyday language usage, from the ancient world to present day. *Qi* has also been utilized in different Chinese philosophical accounts to explore other Chinese key concepts.^3^ However, there has not been a generally accepted or authentic treatise that has integrated different uses of *qi* into a well-ordered theory in Chinese thought. One thing is crystal clear, though: the Chinese metaphysics of *qi* has kept a safe distance from either Cartesian dualism or modern physicalism. As the fundamental elements of the universe, *qi* is understood as neither purely physical nor purely mental. Instead, if one has to understand *qi* in terms of body and mind, one will have to see it as both body and mind, indicating a unique monist view.^4^ Perhaps it can be seen as a special type of neutral monism. While neutral monism usually claims that both mind and body are aspects of a distinct essence of the ultimate reality that is neither mind nor body (Broad, 1925), this view of *qi* holds that *qi* is exactly such a distinct essence, but it is both mind and body.^5^

This special monist view of *qi* has been drawn upon to build the Chinese understanding of organism.^6^ Essential *qi* and original *qi* have been used to account for human life. For one thing, in Confucian tradition, it has been believed that everyone receives essential *qi* from one’s parents to start life. Moreover, some new concepts, such as blood *qi* (血氣, *xueqi*),^7^ have been constructed to address human life (Yang, 2019). Presumably, this type of *qi* must have been developed out of one’s essential *qi*. For classical Confucian masters, the function of one’s blood *qi* must be understood in relation to one’s virtue cultivation. For example, Confucius (551–479 BCE) in the *Analects* (which records Confucius’ words and behavior) remarks about the three stages of human life in relation to one’s blood *qi* in the following way:

There are three things gentlemen should guard against: In youth, their blood *qi is* still unsettled, so the thing to guard against is lust. When they reach their prime, their blood *qi is* full of vigor, so the thing to guard against is being contentious. As they become old, their blood *qi* is declining, so the thing to guard against is being covetous. (Analects 16.7; Ni, 2017, 383)^8^

Although this is not a systematic discourse of *qi*, it discloses that one’s blood *qi* is important to one’s moral life. Specifically, Confucius stresses that one’s *qi* activities must be conducted under the direction of virtue so that one may prevent pitfalls in one’s *qi* development. In other words, the state of one’s blood *qi* is not only fundamental to one’s biological life and health, but is also relevant to one’s moral cultivation and perfection. Thus, a Confucian gentleman’s morality is inseparable from one’s *qi* activities.

This Confucian understanding of moral cultivation in relation to *qi* activities reached its apex in the *Mencius*, a book composed by a brilliant follower of Confucius, Mencius (372–289 BCE). When Mencius was asked by a student about his strong points, he replied, “I am good at cultivating my flood-like *qi* (浩然之氣*hao ran zhi qi*)”:

This is a *qi* which is, in the highest degree, vast and unyielding. Nourishing it with integrity and place no obstacle in its path and it will fill the space between Heaven and Earth. It is a *qi* which unites righteousness and the way. Deprive it of these and it will starve. It is born of accumulated righteousness and cannot be appropriated by anyone through a sporadic show of righteousness. Whenever one acts in a way that falls below the standard set in one’s *xin* (heart/mind), it will starve. (Mencius, 2A:2; Lau, 2003, 63)^9^

Some suggest that Mencius’ flood-like *qi* should not be understood as describing any particular genus of *qi,* such as the blood *qi* that Confucius addresses. Rather, it should be understood as denoting the general concept of *qi*, although it could exist in various types (Li, 2010, 27–8). We think, however, it is more helpful to understand Mencius’ flood-like *qi* as essential *qi*. The point is that for Mencius, one can act with one’s will to cultivate this kind of *qi*, make it prevail in one’s life, and even enable it to carry vast moral and spiritual strength to affect the whole universe. In any case, there is no doubt that both Confucius and Mencius see essential *qi* as the most important constituents of human life, not only filling in and circulating around the body, but also fundamental to the development of one’s moral and spiritual life.

In addition, Xunzi (c. 313–238 BCE), another most important classical Confucian figure, points out,

Fire and water possess *qi* but have no life. Plants and trees possess life, but lack awareness. Birds and beasts have awareness, but lack righteousness. Humans possess *qi*, life and awareness, and add to them righteousness. It is for this reason that they are the noblest beings in the world. (Xunzi, 9.19; Knoblock and Zhang, 1999, 237)^10^

Xunzi is not saying that only things like fire and water possess *qi*, while plants, trees, birds, beasts, and humans possess no *qi* but something else, like awareness or righteousness. In fact, the classical Confucian understanding is that *qi* makes up all different things, including human beings. The difference lies in which type of *qi* is involved. Presumably, since awareness is a distinct and high attribute of life, *qi* for awareness must all be essential *qi*. Indeed, for classical Confucians, it is the different types of *qi* that are constitutive of various things in the universe, showing different properties. For example, a peck of dirt possesses only turbid *qi* (濁氣, *zhuo qi*), whereas a piece of jade has bright *qi* (清氣, *qing qi*); a woman carries more of yin *qi*, whereas a man more of yang *qi*; a genius is invested with high-quality wise *qi* (靈氣, *ling qi*), while a fool possesses only low-quality stupid *qi* (傻氣, *sha qi*).^11^

In short, the classical Chinese idea of *qi* pertains to three dimensions. *Qi* is not only the tiny, invisible components of natural phenomena such as clouds and wind, but is also the ultimate elements of the universe. *Qi* has different types, in which essential *qi* is most powerful and forms life, ghosts, and gods. Finally, *qi* is morally relevant in human life. For Guanzi, essential *qi* can be ensconced by virtue; for Confucius, blood *qi* should be directed by virtue; and for Mencius, flood-like *qi* is “born of accumulated righteousness.” Indeed, the concept of *qi* has generated rich esthetical and ethical connotations in the Chinese language, such as artistic *qi* (氣象, 氣韻 *qi xiang*, *qi yun*), moral *qi* (such as willing *qi* (志氣 *zhi qi*), and courageous *qi* (勇氣 *yong qi*) (Tu, 2001). All things considered, it is only necessary for us to explore the state of essential *qi* involved in a head transplant surgery in order to comprehend the personal identity issues arising from it.

## III. CONFUCIAN MIND AND SOUL: A DIFFERENT PERSON WOULD EMERGE FROM A HEAD TRANSPLANT SURGERY

Who would survive head transplant surgery? To answer this question clearly, we need to look into the Confucian understanding of the heart, mind, brain, head, and soul as well as their relations to essential *qi*. Given that essential *qi* is fundamental to the biological and moral life of a human individual, it is reasonable to infer that this individual’s essential *qi* would determine who he/she is as well as what he/she is. If one’s essential *qi* is all stored in the heart, then the person who provided the body for the surgery would survive the surgery. On the other hand, if it is all placed in the brain, then the person who provided the head would survive the surgery. However, we have reason to believe that neither of these two inferences would be the Confucian conclusion, because one’s essential *qi*, as we will show, is flowing through the entire body. Even if the heart or brain keeps more essential *qi* than other parts of the body, it is still not the case that it alone possesses all essential *qi*. Thus, if we medically create a person by adding a head obtained from person A to a (nonhead) body obtained from person B, the resulting person would definitely have received essential *qi* from both A and B. In this situation, should we recognize this person to be either A or B, both A and B, or neither A nor B? In particular, can we determine whether the heart maintains more essential *qi* than the head, or vice versa, so as to tip the scales in favor of any judgment? Let us turn to the Confucian view of the *xin* (heart/mind) first.^12^

In the aforementioned citation, Mencius remarks that “[w]henever one acts in a way that falls below the standard set in one’s *xin*, [flood-like *qi*] will starve.” Indeed, classical Confucianism developed an advanced idea of *xin*, which inspired tremendous discussions about the function of *xin* (Qiu, 2017). As is well known, Mencius proposed that every human being has four *xin* as four moral germs (or beginnings) to develop Confucian virtues:

The *xin* of compassion is possessed by all humans alike; likewise the *xin* of shame, the *xin* of respect, and the *xin* of right and wrong. The *xin* of compassion pertains to benevolence, the *xin* of shame to righteousness, the *xin* of respect to the observance of the rites, and the *xin* of right and wrong to wisdom. Benevolence, righteousness, observance of the rites, and wisdom do not give me a luster from outside, they are in me originally. (Mencius, 4A:6; Lau, 2003, 247)

Similarly,

Whoever is devoid of the *xin* of compassion is not human, whoever is devoid of the *xin* of shame is not human, whoever is devoid of the *xin* of courtesy and modesty is not human, and whoever is devoid of the *xin* of right and wrong is not human. The *xin* of compassion is the germ of benevolence; the *xin* of shame, of righteousness; the *xin* of courtesy and modesty, of observance of the rites; the *xin* of right and wrong, of wisdom. A human being has these four germs, just as one has four limbs. (Mencius, 2A:6; Lau, 2003, 73)

Thus, Mencius believes that just as everyone has four limbs, everyone is also endowed with four *xin*; namely, the four moral emotions of compassion, shame, respect (or courtesy and modesty), and right and wrong. They constitute the foundations (or germs) of the four cardinal Confucian virtues: benevolence, righteousness, observance of the rites, and wisdom. Obviously, to a modern ear, the *xin* addressed this way is certainly not meant for the heart (as an organ in the body in the modern scientific language), but for the mind. However, Confucianism does not find it necessary sharply to distinguish the mind from the heart. Instead, *xin* is taken to be both physical and mental, just as *qi* is taken to be both material and spiritual. Although Mencius does not specifically point out what kind of *qi* constitutes the *xin*, we can reasonably infer that the *xin* must be composed of the essential *qi* that one originally received from one’s parents, to follow the basic logic of Confucian *qi*. That is, essential *qi* constitutes one’s *xin*, manifesting one’s emotion, perception, and wisdom. Presumably, as different types of *qi* compose different parts of the body, an essential part, such as the *xin*, must be constituted by essential *qi,* while other parts, such as the four limbs, are constituted by non-essential *qi*. To perform its crucial function in human life, essential *qi* must be materially stronger and spiritually more powerful than non-essential *qi*. In this sense, cultivating the *xin* (養心, *yang xin*) and cultivating the *qi* (養氣, *yang qi*) would have the same meaning for Confucianism (cf. Yang, 1999, 161–6).^13^

The Confucian understanding of the endowment of different types of *qi* into different parts of the body as well as the flowing of essential *qi* throughout the body can be tested against the cardinal Chinese medical classic, the *Yellow Emperor’s Canon of Internal Medicine* (皇帝內經, *Huangdi Neijing*), compiled in about the first-century BCE.^14^ According to this classic, one’s essential *qi* has three sources: in addition to its original source from one’s parents, it also comes from the food one eats (穀氣, *gu qi*) and the air one breathes (清氣, *qing qi*). It is stored in the five organs (heart, liver, spleen, lungs, and kidneys) and transforms in the six viscera (stomach, gallbladder, small intestine, large intestine, triple warmer, and bladder) (五臟六腑, *wuzang liufu*). The main function of the five organs, according to this classic, is to maintain essential *qi* to nourish the body, without letting it go out, whereas the main function of the six viscera is to digest essential *qi* and discharge nonessential *qi* out of the body (“discharging without storing”) (*Huangdi Neijing: Suwen* 素問 10, 11; Wu and Wu, 2005, 64–70). In this account, the *xin* is one of the five organs,^15^ but the brain is not. However, the classic indicates that everyone also has five additional vitally functioning organs; namely, the brain, spinal cord, bone, vessel, and womb (for women only), to store one’s essential *qi* (*Huangdi Neijing: Suwen* 11; Wu and Wu, 2005, 69). Accordingly, both the heart and the brain, just as other vital organs, are important parts of the body, keeping one’s essential *qi*.

It is necessary to note that traditional Chinese medicine recognizes a special channel system through which essential *qi* flows throughout the body. According to this theory, there are in total 12 major channels (經絡 *jingluo*), which “connect the organs and viscera inside and link the four limbs and joints outside” (*Huangdi Neijing:* 靈樞 *Lingshu* 33; Wu and Wu, 2005, 650). These major channels, along with their numerous branches, promote the circulation and operation of various types of *qi*, especially essential *qi*, to animate bodily structures, moisten the tendons and bones, and smooth the joints (*Huangdi Neijing: Lingshu* 47; Wu and Wu, 2005, 689). In addition, Chinese physicians have discerned 365 special points on the channels, as special acupoints (穴位, *xuewei*), to conduct acupunctural therapy, in which they use needles to stimulate relevant acupoints to dredge the channels and smooth *qi* circulation (*Huangdi Neijing: Suwen* 58; Wu and Wu, 2005, 256).^16^ In short, this channel system enables various types of *qi*, essential *qi* included, to circulate throughout the entire body, connecting the organs and viscera. Hence, the essential *qi* of a human life is not only located inside a few important organs, but is also dynamically flowing along the channels throughout the body. Accordingly, one’s essential *qi* is by no means restricted only to one’s brain or heart.

Now let us look into the classical Confucian view of the soul to see whether it could also be understood in terms of essential *qi*. In fact, classical Confucianism holds a two-aspect view of the soul: one aspect is called *hun* 魂 (the intelligent soul), and the other is called *po* 魄 (the animal soul).^17^ Interestingly, according to at least one classical view, the *hun* and *po* are inherently related to the *xin:*

Joy in the midst of grief and grief in the midst of joy are signs of a loss of the *xin*. The essential vigor and brightness of the *xin* is what we call the *hun* and the *po* 心之精爽，是謂魂魄. When the *hun* and the *po* leave the *xin*, how can the man continue long? (Zuozhuan: Zhaogong, 25; Legge, 1970, vol. V, 708)

This is to say, the *hun* and *po* are nothing but the principal constituents of the *xin*, which are in turn nothing but essential *qi*. Accordingly, all these subtle human things, *xin*, *hun*, and *po*, are composed of essential *qi* in Confucian beliefs.

When a man is born, [we see] in his first movements what is called *po* (the animal soul) 人生始化曰魄. After this has been produced, it is developed into what is called *hun* (the intelligent soul) 既生魄，陽曰魂. When the use of essential *qi* is multiplied, the *hun* and *po* become strong 用物精多，則魂魄強. They go on in this way, growing in etherealness and brightness, till they become [thoroughly] spiritual and intelligent. When an ordinary man or woman dies a violent death, the *hun* and *po* are still able to keep hanging about men in the shape of an evil apparition; how much more might this be expected in the case of [a powerful person]! (Zuozhuan: Zhaogong, 7; adapted from Legge, 1970, vol. V, 618)

Moreover,

The *hun* is the god nature, and shows that in fullest measure; the *po* is of the ghost nature, and shows that in fullest measure. It is the union of the ghost and god that forms the highest exhibition of [Confucian] doctrine. All the living must die, and dying, return to the ground; this is what is called ghost. The bones and flesh molder below, and, hidden away, become the earth of the fields. But the intelligent soul [the *hun*] issues forth, and is displayed on high in a condition of glorious brightness. The vapors and odors, which produce a sympathetic feeling, are the subtle essences of all things, and a manifestation of the god nature. (Liji: Jiyi; Legge, 1967, vol. II, 220)

Indeed, from a classical Confucian view, when a human being dies, “the *hun qi* returns to heaven; the *po qi* returns to the earth” (Liji: Jiaotesheng; Legge, 1967, vol. I, 444). The *hun qi* is the intelligent soul wandering in heaven as a god, and the *po qi* is the animal soul existing on earth as a ghost. Since individuals originally receive essential *qi* from their respective ancestors, their constituting *qi* is in distinct quality and is, therefore, not similarly strenuous. Accordingly, just as *xin* (heart/mind) is not equally intelligent among people, their *hun* and *po* are not similarly powerful, either. In particular, not everyone’s *hun* and *po* will last for the same length after death: whereas sages’ *hun* and *po* will exist forever, the weakest *hun* and *po* will perish shortly after death.^18^

Further interpretations of the *hun* and *po* are offered in terms of yin *qi* and yang *qi* in a Han-dynasty Confucian document, *Comprehensive Discussions in the White Tiger Hall*, compiled about A.D. 80:

What do the words *hun* and *po* mean? *Hun* expresses the idea of continuous propagation, unresting flight; it is the *qi* of the Lesser Yang, working in a human being in an external direction, and it governs the nature.

*Po* expresses the idea of a continuous pressing urge on a human being; it is the *qi* of the Lesser Yin, and works in this human, governing the emotions.

*Hun* is connected with the idea of “weeding,” for with the instincts the evil weeds (in human nature) are removed.

*Po* is connected with the idea of “brightening,” for with the emotions the interior (of the personality) is governed.^19^

Here yin *qi* and yang *qi* can be understood as two types of essential *qi.* Some Han-dynasty texts even hold that everyone has three *hun* and seven *po* (Needham and Tsien, 1985, 83). It is a bit difficult to ascertain the reason for these numbers, given that the five organs and six viscera have been set in Chinese medicine, as shown above. Presumably, three *hun* may be connected with the three relational bonds—ruler-ruled, parent-child, and husband-wife—that Confucianism has underlined as the most important unities in the human world. Seven *po* may be set in relation to the sensory-perceptive quality of *po*, as there are seven inlets or openings on the head (七竅 *qi qiao*: ears, eyes, nostrils, and mouth). In addition, they may also be correlated with the seven emotions (七情 *qi qing*: joy, anger, grief, fear, love, hate, and desire) that are supposed to be governed by *po* (Needham and Tsien, 1985, 83). In any case, we have no reason to doubt that three *hun* and seven *po* are also composed of essential *qi*.

Taken together, no matter whether we talk about one *hun* and one *po*, or three *hun* and seven *po*, it is clear that *hun* and *po*, as the essential vigor and brightness of the *xin*, are simply the essential *qi*, which is the principal constituent of human life. Just as we cannot believe that *xin* is all placed in the heart or brain, neither can we infer that *hun* and *po* are all placed in the heart or brain. It would be a silly joke for a Confucian scholar to suggest that one’s soul is located in the pineal gland in the brain, as Descartes believed.^20^ Rather, as essential and vital *qi* which is both material and spiritual, the *xin*, *hun*, and *po* must be understood as existing in every important part of the body, particularly in the vital organs and the channels. Although one’s heart and brain belong to such vital organs, neither is uniquely special or crucial in containing one’s *xin*, *hun*, and *po* entirely so as to determine one’s personal identity. Accordingly, after a head transplant surgery, the person (C) who emerges from it would definitely carry part of the *xin*, *hun*, and *po* (namely, essential *qi*) from each of the two persons, A and B, who provided the head and the body, respectively.

As a result, this person, C, cannot be taken as either A or B. Even if A and B are of the same sex, say two women, C would still be neither A (who provided the head) nor B (who provided the body), because C’s essential *qi* is similarly from both of them. Some may contend that Confucians should have more reason to believe that C would be B because the body contains more vital organs (thereby more essential *qi*) than the head. If a person remains the same person after a heart transplant, they may contend, a person should remain the same person after a head transplant, because changing one vital organ (either the heart or the brain) does not change much of essential *qi* that one carries. We think this contention is misguided for two reasons. First, the head by no means contains only the brain. It also has seven inlets (namely ears, eyes, nostrils, and mouth, as mentioned above) in it. These inlets, from the Confucian perspective, are vitalized by a person’s essential *qi* and play crucial functions for that person’s life. Moreover, the head keeps the converging points of the several major channels in which a person’s essential *qi* goes through, so that the head may reasonably store no less essential *qi* than the body. Accordingly, the head should not be taken as only one of the major organs (such as the heart) which can be transplanted without significantly changing a person’s essential *qi*. Rather, if one’s head is changed, the significant amount of one’s essential *qi* is also changed, so that one cannot remain the same person as before.

Is it possible for a conjoined A and B to survive head transplant surgery? In this case, C would remain the two original persons, A and B, conjoined, much like conjoined twins who are born physically connected to each other. That is, although A and B will remain physically connected after the transplant surgery, they are actually two different individuals. However, if this is the case, it would mean that their essential *qi* is not mixed with each other. That is, A’s essential *qi* is still located only in the head, and B’s essential *qi* only in the body, although they are connected to each other through surgery. This is impossible given the existence of the channel system that Confucian medicine has disclosed for any living organism: essential *qi* must flow through the head and the body along the channels. If essential *qi* cannot flow this way, there would be no living human organism. On the other hand, that two conjoined individuals could survive is because they each maintain a roughly independent channel system through their head and body, even if their bodies are in part connected—most often at the abdomen or pelvis. Thus, successful head transplant surgery must result in the mixed essential *qi* of A and B rather than the unmixed essential *qi* of two conjoined persons.

Finally, is it possible to consider C to be both A and B in one life since C carries the mixed essential *qi* of both A and B? We believe this is also impossible. From a Confucian perspective, a person’s *xin*, *hun*, and *po* in the quality of essential *qi* must make a united personality in one life that cannot be shared by another person. This is like the analogy of natural reproduction: by combining the essential *qi* of the father and the mother, the child procreated is a new, different individual. Thus, Confucians can only identify C as a new person with a personal identity differing from either A or B. For those who do not think the Confucian resources of essential *qi* as we have provided can sufficiently lead to this conclusion, in the following section we explore the Confucian relationalistic view of personal identity to strengthen our argument. We will show that in the Confucian relationalist lifeworld, not only is it impossible for C to be either A or B, but it is also impossible for C to be both A and B in one life.

## IV. CONFUCIAN FAMILIAL RELATIONALISM: NO EXISTING PERSON CAN SURVIVE A HEAD TRANSPLANT

In addition to the fundamental importance of essential *qi* for personal identity, one’s personal identity is also socially determined in the Confucian lifeworld. In this world, no one can define himself or herself in isolation from one’s social relations, especially family relations. In this section, we will first illustrate this relationalist view by introducing a Chinese legendary story of a head transplant and then explore the implications of this view for the issue of personal identity in head transplantation.

This story occurs in a traditional novel titled, *Strange Tales from Make-Do Studio* (聊齋志異), constructed by a popular Chinese author, Pu Songling (蒲松齡, 1640–1715).^21^ It goes like this. A local Confucian scholar named Zhu Erdan became the good friend of judge Lu, a powerful judge from the after-death underground world. Judge Lu wanted to help Zhu become a successful scholar by changing his heart. He managed to find a brilliant heart from one of the tens of thousands of dead bodies and transplanted it into Zhu’s chest to replace his original heart. Zhu acquired great improvement in memory and writing ability. Meanwhile, Zhu did not find the face of his wife appealing, although he was happy with the lower parts of her body. He asked Judge Lu to change her facial features. Judge Lu replied with a laugh and requested some time to work out the details. Eventually, he obtained the head of a beautiful woman and successfully conducted a head transplant surgery for Zhu’s wife. She was thus changed into a beautiful woman, and Zhu rejoiced. However, the head had been acquired from a local official couple’s daughter, who had been murdered by a robber. After the official couple discovered that their daughter’s head was fitted on the neck of Zhu’s wife, they happily accepted Zhu’s wife as their daughter and accepted Zhu as their son-in-law. The original head of Zhu’s wife was put together with that daughter’s corpse and buried.

This story integrates Buddhist and Daoist religious beliefs, such as the existence of an after-death underground world in which there are judges who rule, and that such judges can even come to this world and intervene with living human affairs.^22^ In addition, the story is entangled with a series of mistaken ideas. First, it assumes that one’s intelligence and thought are associated with the heart rather than the brain (at least in the case of Zhu). Second, it holds that even if one’s head is changed, one’s personal identity will remain the same (in the case of Zhu’s wife). Finally, it suggests that two persons can survive in one individual after a head (or whole body) transplant is conducted (in the case of Zhu’s wife and the official couple’s daughter). The first misunderstanding aside, we argue that the two latter ideas are impossible in the real Confucian life-world in which people hold the Confucian relationalist view of personal identity. We shall draw on Confucian family-based intellectual resources to show that one’s relationalist features and family relations constitute a necessary condition for understanding or determining who one is. In short, in this case, neither Zhu’s wife, nor the official couple’s daughter, let alone both of them, could survive a head/whole body transplant surgery to continue life.

Although classical Confucian figures never directly addressed the relationalist nature of individual identity, we can reasonably reconstruct their view. Individualism holds that one’s personal identity is determined completely by one’s individualist traits, such as the continuity of an immaterial substance (René Descartes), the continuity of one’s mentality (John Locke), or the continuity of one’s body (naturalist science). That is, one’s identity can be defined solely in terms of one’s individual features, independent of one’s relationships with other individuals, such as one’s immediate family members. In contrast, Confucianism holds a relationalist view: one’s personal identity cannot be established without reference to the essential relationships with one’s immediate family members.^23^ That is, everyone has to be put into a suitable place and play a proper role in the family (such as son or daughter, husband or wife, father or mother) to obtain a clear understanding of that person’s identity. For example, an orphan is not a child without biological parents, but is a child who has lost his/her biological parents, who will still serve as a necessary background for others to understand who he/she is (e.g., “they died when he/she was one year old”). In short, Confucianism espouses a kind of relationalism: human individuals are relational individuals, and the identity of an individual cannot be established without having his/her social relations, especially familial relations, appreciated.

In the Confucian lifeworld, individual identity is recognized, manifested, and realized in human relations through ritual performance. Confucian rituals are a series of behavior patterns for harmonizing individual interactions in learning and exercising the Confucian virtues (Fan, 2010a). These rituals, especially the funeral and sacrificial rites of the family, bring the ancestors and descendants together to accomplish the spiritual unity of the entire family (Fan, 2010b). From the perspective of Confucian cosmogony and cosmology, individual life is taken to be a gift from one’s ancestors, especially one’s parents, under a natural order mandated by Heaven:

Heaven and earth existing, all things then got their existence. All things having existence, afterwards there came male and female. From the existence of male and female there came afterwards husband and wife. From husband and wife there came parent and child. From parent and child there came ruler and minister. From ruler and minister there came high and low. When [the distinction of] high and low had existence, there came rituals and righteousness. (*Yijing: Xugua*; adapted from Legge, 1973, 435–6)

This perspective indicates not merely the Confucian understanding of the genealogy of natural world and human society. It also sets forth the central relational attributes of human persons in determining personal identity while also alluding to proper moral norms in guiding personal actions. It is well known that Confucian tradition underscores the cardinal importance of the family in cultivating one’s virtuous character, as well as accentuates one’s familial roles in shaping one’s identity (Fan, 2015; Fan and Wang, 2015). Based on such Confucian resources, it is no difficulty to summarize a few necessary relationalist features, to which Confucian people normally appeal in determining individual identity. By “relationalist features” we mean those individual features that are shared by an individual with his/her familial relatives so as to place him/her into the web of family relations which make up his/her identity. For example, given that the parent–child relationship is essential for identifying an individual, a child needs to share some relationalist features with his/her parents, such as the same DNA, similar bodily characteristics (such as facial features), similar temperament, and/or common life experience, in order to establish the parent–child relationship with them and to determine his/her personal identity. The same will be true with one’s adoptive parents (if one is an orphan) or other family members, although in a less strong or complete degree of sharing of such relationalist features than a biological parent.^24^ If an individual lacks such relationalist features, then he/she will not be able to form important family relationships, and his/her personal identity will be difficult to determine in Confucian society.

One’s relationalist features constitute a necessary condition for establishing one’s family relations and determining who one is in Confucian society. The person who emerges out of head transplant surgery cannot be put into the web of family relations without enormous contradictions because this person lacks the proper relationalist features for such relations. Let us go back to the above novelistic story. First, after Judge Lu performed the head transplant surgery, would the woman be Mr. Zhu’s original wife (namely, the woman who provided the body)? In the Confucian lifeworld, this would be impossible. Even if she herself believed so (and this is quite unlikely, given the change of the head), other relatives would hardly accept it because she now has dramatically different facial features from those of Zhu’s original wife with which they have been familiar. She would also obtain quite different senses, ideas, and preferences from those of Zhu’s original wife. The original wife’s biological parents, if available, would find a quite different “daughter,” and Zhu’s parents would find a totally new “daughter-in-law.” The children of Zhu and his original wife, if any, would not find her to be their original mother. If this woman has additional children with Zhu, they will resemble Zhu’s original wife rather than her. In short, even if Zhu would be willing to take this woman as if she were his original wife (because he loves her new face), she will still lack the necessary relationalist features for that personal identity.

On the other hand, would the woman after surgery be the official couple’s daughter (namely, the girl who provided the head)? This would also be impossible. Even if the official couple were willing to accept this identification, the biological parents of Zhu’s original wife would reject it because they would not be able to accept that their daughter had disappeared or changed into the official couple’s daughter. This may be why the story does not mention the existence of the biological parents of Zhu’s wife at all: it has to play this trick to avoid the personal identity difficulty. The DNA genes contained in this new woman’s reproductive cells would be the same as the genes of Zhu’s original wife. If they have additional children, their DNA will be similar to the genes of Zhu’s original wife rather than to the official couple’s daughter. Finally, if it happened that any man had fallen in love with the official couple’s daughter before she died, he would surely not accept that she had now become Zhu’s wife.

Could this woman be both Zhu’s original wife and the official couple’s daughter at the same time? We think this is unconceivable in the Confucian relationalist lifeworld, even if this woman came to believe that she is two-persons in one (which is unlikely). She may possibly believe so for a moment, but it will be extremely difficult for her to believe it for long. She cannot behave as two persons in everyday life contexts, and there is no way for her relatives to treat her as both Zhu’s original wife and the official couple’s daughter. Is it possible for her to be these two persons in the sense that she is Zhu’s original wife for some relatives (such as Zhu and his parents) whereas she is the official couple’s daughter for other relatives (such as the official couple)? We think this is also beyond the pale because the two different accounts of her identity held by two different groups of relatives would generate enormous confusions and crises in her life contexts. Finally, is it possible for this woman to be Zhu’s original wife sometimes, and the official couple’s daughter at other times? For example, she may be Zhu’s original wife at home, but the official couple’s daughter when she goes out. Again, we think this is impossible, because it would involve intractable relation conflicts among this woman, Zhu, and other relatives for understanding and establishing her identity.

In sum, the imagined hybrid woman (formed by Judge Lu through combining the body of Zhu’s wife and the head of the official couple’s daughter) would be neither Zhu’s original wife nor the official couple’s daughter. It would also be impossible for her to be both Zhu’s wife and the official couple’s daughter living in one individual. No living person could survive a head/whole body transplant surgery in the Confucian lifeworld because everyone’s personal identity is socially established, which cannot be maintained through such a transplant. One will not possess the relationalist features necessary to inherit any legitimate personal identity.

## V. CONCLUDING REMARKS

This essay argues that the Confucian tradition offers useful intellectual resources to deal with personal identity issues arising from the possible technology of head transplants. It is helpful to draw on classical Confucian ideas to expand our philosophical imagination. This Confucian perspective differs from dominant contemporary views and carries significant ethical implications. In particular, given that a head transplant surgery would rip apart relationalist features, destroy family relations, and mess up personal identity, we have good reason to hold that such surgeries are morally inappropriate. We should not conduct such surgeries, even if they become technologically possible.

## Footnotes

^1.^

*Qi* (氣) has multiple English translations in the literature, including air, breath, vapor, stream, vital fluid, energy, ether, material force, prime force, vital force, and subtle spirits (Onozawa, Fukunaga, and Yamai, 1999, 534–5). This article simply uses pinyin *qi* to capture this traditional Chinese concept.

^2.^
This English translation is our own. For a Chinese version of the book, see Guanzi (2000).

^3.^
For example, Neo-Confucian scholar Zhu Xi (1130–1200) worked out a new, sophisticated account of *qi* in relation to *li* (principle). Although he holds that *qi* and *li* are always intertwined with each other, he also insists that *li*, rather than *qi*, represents the metaphysical Dao of the universe. Hence, *li* radically differs from *qi* in his account. As a result, his account cannot refrain from a sense of dualism. We think his account has deviated from the classical view of *qi*. In this article, we primarily draw on classical rather than Neo-Confucian resources to construct our arguments.

^4.^
This uniquely monistic nature of *qi* may have been held similarly by both classical Confucians and Daoists. Their disagreement lies elsewhere. Although Daoists believe that human bodily immortality is pursuable through Daoist *qi* exercise, temple liturgy, and alchemical techniques (Zhang, 1994, 1996; Tu, 2001; Zhong, 2016), Confucians hold that human bodily death is inevitable. Confucians dedicate themselves to the cultivation of moral *qi* and participation in proper rituals in treating both living persons and the souls of the dead appropriately (Zhou, 2005; Yang, 1999; Yang and Zhu, 2005).

^5.^
It is not easy to offer an explicit explanation regarding how *qi*, as the fundamental elements of the universe, can be both physical and mental. This classical Chinese understanding has both similarities to and differences from ancient Greek philosophical views. For example, *qi* is similar to the atoms in Democritus’ atomism in that both atoms and *qi* are very small, invisible particles in various types, making up myriad things in the world. But they differ in that atoms are purely material while *qi* is both material and mental. Moreover, *qi* differs from Plato’s “forms” since the forms exist as the timeless ideal universals of which this-worldly objects are the mere imitations, whereas the various types of *qi* themselves exist and transform in this world. Finally, the Chinese notion of *qi* may be most similar to Aristotle’s notion of *minima naturalia* (namely, the smallest parts of a homogenous natural substance). Such “natural minimum,” in Aristotle’s hylomorphic metaphysics, is a compound of material matter and an immaterial substantial form that imparts its essential nature and structure. However, *qi* has never been expressed as a combination of matter and form. In addition, although there are different kinds of natural minimum for different substances, Aristotle has never developed a notion of anything like the “essential *qi*” that can not only unite with each other, but can also develop and transform by itself, as in the Chinese metaphysics of *qi*. For a comparison between Aristotle’s view of the soul as substantial form and the Confucian view of the soul as essential *qi*, see footnote 18.

^6.^
As noted by Joseph Needham, “in accord with the character of all Chinese thought, the human organism was an organism, neither purely spiritual in nature nor purely material. It was not a *machina* with a single *deus* in it, which could go off and survive somewhere else . . . the line drawn between spirit and matter in all Chinese thinking was extremely vague . . . If one can say that there was a complete continuity, in the Chinese philosophical outlook, between “something” and “nothing,” “solidity” and “emptiness,” why should one be surprised that there was also continuity between “matter” and “spirit?” *Chhi* [*qi*], one could say, was on the borderline” (Needham and Tsien, 1985, 92–3). In addition, contemporary Chinese scholars investigating traditional Chinese medicine have attempted to understand the dual-property (both mind and body) of *qi* in terms of modern scientific discoveries, especially those in quantum mechanics. In particular, they tend to believe that *qi* exists like quantum particles (as the latter generate such puzzling phenomena as wave-particle duality and quantum entanglement that seem to suggest that the particles have the attributes of both body and mind). See, e.g., Qu (2014). We do not explore this type of understanding in this article.

^7.^
Blood *qi* had become a popular concept regarding human life in Confucius’ time. See Peng (2019).

^8.^
Our citations of the *Analects* are all adapted from Ni (2017).

^9.^
Our citations of Mencius are all adapted from Lau (2003).

^10.^
Our citations of Xunzi are all adapted from Knoblock and Zhang (1999).

^11.^
This classical Confucian view of *qi* as endowed differently in distinct objects and individuals was also fully supported and strengthened by later Confucians, such as some premodern Confucian scholars, like Dai Zhen (1724–77) (Onozawa, Fukunaga, and Yamai, 1999, 452–66).

^12.^
The *xin* (心) is usually translated into English as “heart/mind.”

^13.^
We are fully aware that this is a controversial claim. Some Confucian scholars do not accept the metaphysical existence of *qi*. For them, *qi* is only phenomenal, whereas *xin* or *li* (order) possesses metaphysical meaning independent of *qi*. We think their view is suspicious of dualism, and this article works to reconstruct a Confucian monistic view in terms of *qi*. We thank Fang Xudong and Tang Wenming for discussing these issues with us.

^14.^
It is generally taken that this medical classic was worked out in accordance with basic Confucian and Daoist principles (Wu and Wu, 2005). So, it is reasonable to draw on its intellectual resources to construct our Confucian arguments regarding the personal identity issues at stake.

^15.^
Throughout the *Yellow Emperor’s Canon of Internal Medicine*, there are many passages in which the importance of the *xin* is underscored. For instance, it is claimed that “*xin* is the base of life and the place where the spirit locates” (*Huangdi Neijing: Suwen* 9; Wu and Wu, 2005, 62); “among the five organs and six viscera, *xin* is the dominator” (*Huangdi Neijing: Lingshu* 36; Wu and Wu, 2005, 658); and “*xin* is the place where the spirit situates” (*Huangdi Neijing: Lingshu* 80; Wu and Wu, 2005, 822). Nevertheless, *xin* remains only one of the important organs in storing essential *qi*. Indeed, following the view of this classic work, Chinese people never think that one would become a different person after a heart transplant, just as one would remain the same person after a liver or lung transplant.

^16.^
Under “scientific” guidance, modern Chinese researchers concentrated their efforts on attempting to discover a concrete tubing structure (like nerve fibers or blood vessels) below the specified routes of the channels in order to affirm the “science” of the channel system. That is, they tried to find an observable anatomical structure or particular physicalist basis to ground the channel phenomenon on a purely materialist foundation. Although enormous labor and energy were invested, all was done in vain. The truth of the matter is that the channel phenomenon is a special sensational phenomenon felt by individuals and discovered by physicians over 2000 years ago. The channel account, established in relation to the classical account of *qi*, cannot and should not be rejected in light of modern scientific theory (Fan, 2003).

^17.^
According to historic studies (Yu, 1987), this two-aspect view of the soul has long been a typical Chinese understanding. The indigenous Chinese tradition held this view along with belief in the afterlife long before Buddhism was imported into China in the first-century BCE.

^18.^
It is worth noting that this Confucian view of the soul in terms of essential *qi* significantly differs from Aristotle’s view of the soul in terms of substantial form, although both views, at first glance, appear to be similar with respect to the differences in types of *qi*/form for nutritive, appetitive, sensory, locomotive, and intelligent organisms. First, the Confucian soul is essential *qi*, which is tiny elements and should be understood as both matter and form, while Aristotle’s soul is defined as form only. Second, Confucians cannot use the analogy of geometric figures to understand the soul as Aristotle can. Finally, the soul still exits after death in the Confucian account, while in Aristotle’s account, the soul dissolves at death. I am grateful to Jason Eberl for discussing these issues with me.

^19.^
Our English translation of this passage is adapted from Needham and Tsien (1985, 87).

^20.^
It is worth noting that at least in one chapter of the *Yellow Emperor’s Canon of Internal Medicine,* we read that “the liver . . . is the place where the *hun* lies” (*Huangdi Neijing: Suwen* 9; Wu and Wu, 2005, 63). However, taking the classic as a whole, we should not understand this as saying that the *hun* is only or always located in the liver. The *hun* is just a group of essential *qi*. It is the general view of the classic that essential *qi is* stored in all important organs and flowing through the channels. Just as we cannot take essential *qi* as stored only in the heart, neither can we take it as stored only in the liver.

^21.^
For an English version of the story, see Mair and Mair (1996, 61–72).

^22.^
Both Chinese Buddhism and Daoism developed such religious beliefs on an after-death underground world in the early ages of China, most probably before the starting of the Tang dynasty in 618 (see, e.g., Xiao, 2006). I am grateful to Ellen Zhang for offering this reference.

^23.^
It is necessary to note that relationalism differs from individualism in defining human identity, but it is not necessarily anti-substantivism. As Joseph Kaipayil puts it clearly, “Relationalism is not anti-substantivism. On an anti-substantivist view, things are not objects in their own right, but only events dependent on other events for their existence. Even if we grant the argument that relations are ontologically more fundamental than entities themselves, the question is, if there are not entities with some enduring substantivity, how do relations themselves exist? Relation is “holding” between two or more things. If entities disappear, relations will also disappear” (Kaipayil, 2009, 8).

^24.^
For example, an orphan will need the relationalist features of shared life experiences with his/her adoptive parents or others who brought him/her up in order to establish his/her personal identity.
